# A Case Report: Genetically Distinct Severe Acute Respiratory Syndrome Coronavirus-2 Variant Causing Reinfection

**DOI:** 10.3389/fmicb.2021.792514

**Published:** 2021-12-09

**Authors:** Mohammad Enayet Hossain, Md. Mahfuzur Rahman, Md. Shaheen Alam, Monira Sarmin, Yeasir Karim, Mehedi Hasan, Ananya Ferdous Hoque, Md. Mahmudul Hasan, Mohammed Ziaur Rahman, Mohammod Jobayer Chisti, Mustafizur Rahman

**Affiliations:** International Centre for Diarrhoeal Disease Research, Bangladesh (icddr,b), Dhaka, Bangladesh

**Keywords:** SARS-CoV-2, reinfection, variant, COVID-19, Bangladesh

## Abstract

**Background:** The emergence of novel variants has been a great deal of international concern since the recently published data suggest that previous infections with SARS-CoV-2 may not protect an individual from new variants. We report a patient had two distinct episodes of COVID-19 with different variants of SARS-CoV-2.

**Methods:** The nasopharyngeal samples collected from the two episodes were subjected to whole-genome sequencing and comparative genome analysis.

**Results:** The first infection presented with mild symptoms, while the second infection presented with severe outcomes which occurred 74 days after the patient recovered from the first episode. He had elevated C-reactive protein, ferritin, and bilateral consolidation as a sign of acute infection. Genome analysis revealed that the strains from the first and second episodes belonged to two distinct Nexstrain clades 20B and 20I and Pangolin lineages B.1.1.25 and B.1.1.7, respectively. A total of 36 mutations were observed in the episode-2 strain when compared with the reference strain Wuhan-Hu-1. Among them, eight mutations were identified in the receptor-binding domain (RBD).

**Conclusion:** Our findings concern whether the immunity acquired by natural infection or mass vaccination could confer adequate protection against the constantly evolving SARS-CoV-2. Therefore, continuous monitoring of genetic variations of SARS-CoV-2 strains is crucial for interventions such as vaccine and drug designs, treatment using monoclonal antibodies, and patient management.

## Introduction

The ongoing coronavirus disease 2019 (COVID-19) pandemic caused by the severe acute respiratory syndrome coronavirus-2 (SARS-CoV-2) has severely affected the global health and healthcare system of many countries. As of October 30, 2021, more than 246 million cases of COVID-19 and 5 million associated deaths have been recorded globally^1^. SARS-CoV-2 has a 30-kb positive sense single-stranded RNA genome that codes for 16 non-structural proteins (NSP1–NSP16), 4 structural proteins [spike (S), envelope (E), membrane (M), and nucleocapsid (N)], and 9 accessory factors (Gordon et al., 2020). The S glycoprotein of SARS-CoV-2 is a common target for potential drug and vaccine design as it initiates viral entry into host cells by attachment to angiotensin-converting enzyme 2 (ACE2) receptors (Hatmal et al., 2020). Frequent mutations in the genes encoding the S protein contribute significantly to SARS-CoV-2 evolution and transmission. The major globally circulating SARS-CoV-2 variants of concern (VOC) identified are Alpha (B.1.1.7), Beta (B.1.351), Gamma (P.1), and Delta (B.1.617). These variants have a common mutation N501Y in the S protein receptor-binding domain (RBD), except the Delta variant, which results in the greater affinity of their S protein for ACE2, enhancing viral attachment and subsequent entry into host cells (Kemp et al., 2020; Barton and MacGowan, 2021). The emergence of the novel variants has been particularly of great concern (Rahman et al., 2021) since recently published data suggest that previous infections with SARS-CoV-2 may not protect an individual from new variants of SARS-CoV-2 giving rise to reinfection cases in the community (Fonseca et al., 2021). Therefore, it is important to elucidate the genetic information of SARS-CoV-2 cases for clinical and therapeutic management.

The current report describes two distinct episodes of COVID-19 in a 52-year-old hypertensive male physician with different variants of SARS-CoV-2.

## Case Details

On November 28, 2020, the case presented with low-grade fever, cough, and sore throat and was diagnosed positive for SARS-CoV-2 by real-time reverse-transcription polymerase chain reaction (RT-PCR). Real-time RT-PCR reactions were performed using iTaq Universal Probes One-step Kit (Bio-Rad Laboratories, CA, United States) in the CFX96 Touch Real-time PCR Detection System (Bio-Rad Laboratories, CA, United States). RT-PCR targeted the RdRp (ORF1ab) and N genes as per the protocol described by the Chinese Center for Disease Control and Prevention (China CDC) and recommended by the World Health Organization (WHO) (CDC, 2020; World Health Organization [WHO], 2020b). This RT-PCR method has a sensitivity of 98.2% and specificity of 100% compared to different real-time RT-PCR assays (Vogels et al., 2020; Chung et al., 2021). The patient did not require hospitalization, and after 7 days, his symptoms went away, though he experienced fatigue for two more weeks. Since his symptoms were mild, he did not require immunosuppressive steroid drugs for recovery. He was retested on December 11, 2020, was diagnosed negative for SARS-CoV-2 by RT-PCR, and returned to routine work in the hospital. After 12 weeks from the first infection, he presented 2-day fever (highest recorded temperature was 101°F), cough, and sore throat and was diagnosed as SARS-CoV-2 positive by RT-PCR on February 23, 2021. He already had taken his first shot of the Oxford–AstraZeneca vaccine (Covishield) on February 20, 2021, but completed the second dose later on May 30, 2021. After 3 days of stay at home, on February 26, he was hospitalized at the Dhaka Hospital of icddr,b for generalized weakness and lightheadedness with one episode of syncopal attack. He was clinically stable except for low blood pressure. In the meantime, laboratory investigations revealed absolute lymphopenia [absolute lymphocyte count 0.53 × 10^9^/L (reference: 1.5–4.0 × 10^9^/L)] and thrombocytopenia [platelet count 124 × 10^9^/L (reference: 150–450 × 10^9^/L)] with hypocalcemia [calcium 2.05 mmol/L (reference: 2.2–2.65 mmol/L)], hyponatremia [sodium 124.65 mmol/L (reference: 135.0–145.0 mmol/L)], hypokalemia [potassium 3.47 mmol/L (reference: 3.5–5.3 mmol/L)], and raised inflammatory markers [C-reactive protein (CRP) 2.43 mg/dl (reference: < 0.5 mg/dl), lactate dehydrogenase (LDH) 633.8 U/L (< 248 U/L), ferritin 4,515.27 ng/ml (21.18–274.66 ng/ml)], raised transaminase (4× ULM), and normal D dimer [400 ng/ml (reference: < 500 ng/ml)] and fibrinogen (245.3 mg/dl (reference: 180–350 mg/dl)]. He was managed by following standard hospital guidelines (injected ceftriaxone and ciprofloxacin, subcutaneous low molecular heparin, ivermectin, zinc, pantoprazole, and vitamins D and C) on the management of COVID-19 patients, which is evidence-based and almost aligned with WHO and national guidelines (World Health Organization [WHO], 2020a; Ahmed et al., 2021). A chest x-ray (CxR) posteroanterior view revealed bilateral consolidation with a score of 3 out of 8 (where 0 means no involvement and 8 means involvement of both lungs entirely). On the second day of admission, his clinical condition deteriorated. He had developed hypoxemia (SpO2 < 90% in room air), and WHO standard low-flow oxygen therapy (2 L/min) was added using a nasal cannula. His repeated CxR showed bilateral consolidation with a score of 6 out of 8. His antibiotics were switched to intravenous meropenam plus, tigecycline, remdesivir, dexamethasone (Government of the People’s Republic of Bangladesh, 2020), and tab baricitnib (Kalil et al., 2021).

**FIGURE 1:**
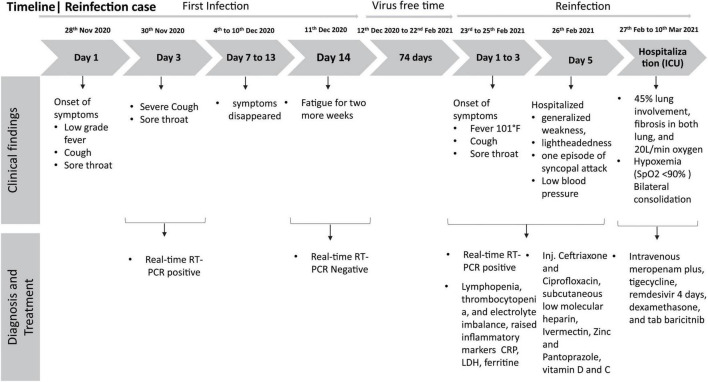
Timeline of the reinfection case.

Repeat lab test findings, as shown in Supplementary Table 1, revealed neutrophilia with lymphopenia with thrombocytopenia, transaminase further raised to 9× ULM, and inflammatory markers except CRP raised further [CRP 0.86 mg/dl, LDH 781.5 U/L, ferritin 11833.74 ng/ml]; however, hepatitis B and C were negative. By the next day, his oxygen demand increased to 5 L O_2_/min to maintain desired saturation. On that very day, he was transferred to the intensive care unit (ICU) of Evercare Hospital Dhaka and was treated with high-flow nasal cannula (HFNC) having oxygen flow at 20 L/min at FiO_2_ 60%. A high-resolution computed tomography (HRCT) chest scan revealed ground-glass opacity in the left upper lobe and middle lobe indicating a pattern of acute viral pneumonia typical of COVID-19 with 45% lung involvement and also fibrosis was seen in the lower zone of both lungs with septal thickening. Remdesivir was discontinued after 4 days of initiation for high transaminase. A diagnosis of syndrome of inappropriate antidiuretic hormone secretion (SIADH) was made for low serum sodium (simultaneously with high urine sodium and osmolality and low serum osmolality) which was managed with the moderation of fluid intake with sodium supplementation. After 12 days in the ICU, his condition improved gradually; oxygen therapy was titrated to 5 L/min with a nasal cannula. His inflammatory markers gradually went down; transaminases also touched the baseline, except persistent hyponatremia and hypokalemia with mild improvement of the CxR. Oxygen was discontinued on the 20th day of admission as he was maintaining the desired saturation with room air. He was clinically and hemodynamically stable and was discharged on that day (Figure 1).

**FIGURE 2:**
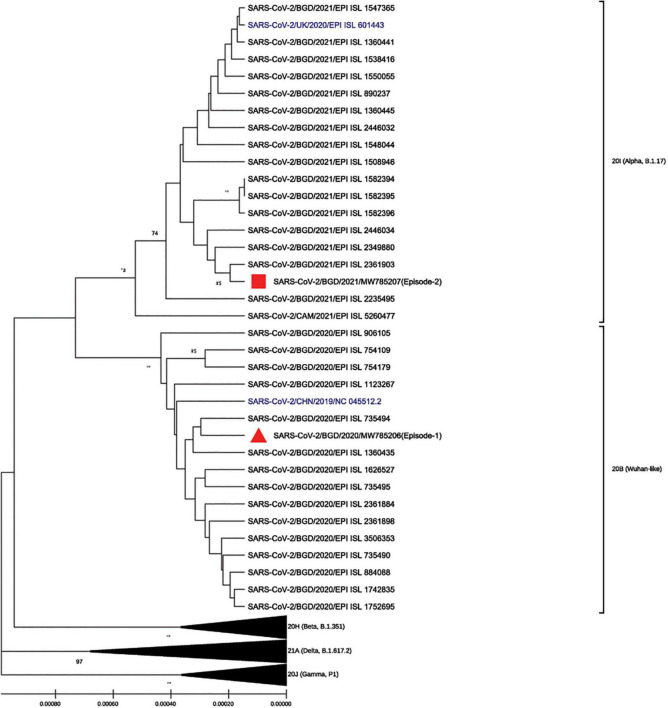
Phylogenic analysis of the strains isolated from episode-1 and episode-2 with globally circulating SARS-CoV-2 strain and reference Wuhan-Hu-1 and Alpha strains (in blue) using MEGA-X (version 10.0.5) software and ClustalW for multiple sequences alignment in the BioEdit v7.2 program. The phylogenetic tree was constructed with 1,000 bootstrap replications using the UPGMA method, and evolutionary distances were computed using the Kimura 2-parameter. Bootstrap values lower than 70 are not shown.

To better understand the genetic makeup of the viruses and rule out the possibility of relapse, full genome sequencing of the viruses from the two episodes was conducted. Viral RNA was extracted using QiaAmp Viral RNA Mini kit (Qiagen, Hilden, Germany) from patient nasopharyngeal samples from both episodes and was subjected to cDNA synthesis using the iScript™ cDNA synthesis kit (Bio-Rad Laboratories, CA, United States). The gene segments were amplified separately by using the GoTaq G2 Hot Start Taq polymerase kit (Promega Corp., WI, United States) with specific primer sets (Quick, 2020) to complete the whole genome of SARS-CoV-2. PCR products were visualized through gel electrophoresis (1.5% agarose) and were purified using the ExoSAP-IT PCR cleanup kit (Affymetrix, California, United States). The cycle sequencing reaction was performed using the ABI BigDye Terminator v.3.1 cycle sequencing kit (Applied Biosystems, United States). Sanger sequencing was carried out in the ABI 3500 XL genetic analyzer (Applied Biosystems, Foster City, United States) using the forward and reverse primers separately. The chromatogram nucleotide sequences were inspected using Chromas v2.23 (Technelysium, QLD, Australia), and the consensus sequences were assembled using SeqMan II (DNASTAR, WI, United States). Multiple sequence alignment was performed using the BioEdit v7.2 program (Hall, 1999) with the built-in ClustalW feature. The genome sequences from episode-1 (GenBank accession number MW785206, GISAID accession number EPI_ISL_5540133, GC content, 38%) and episode-2 (GenBank accession number MW785207, GISAID accession number EPI_ISL_5540134, GC content, 38%) were aligned with the reference strain Wuhan-Hu-1 (GenBank accession number NC_045512). The phylogenetic analysis was performed with globally circulating SARS-CoV-2 reference sequences using MEGA-X (version 10.0.5) software (Figure 2). The phylogenetic tree was constructed with 1,000 bootstrap replications using the UPGMA method. The evolutionary distances were computed using the Kimura 2-parameter. The sequencing results revealed that the two independent infections were caused by two different variants. According to clade-based analysis, the strains from the first and second episodes belonged to the GISAID clade GR. Phylogenetic analysis showed that the lineage B.1.1.25 was detected in the first episode, whereas the lineage B.1.1.7 was detected in the second episode according to the Pangolin lineage classification.^2^ According to the Nextstrain classification, the strains from the first and second episodes were placed in clades 20B and 20I, respectively (Figure 2). A comparison between the first (episode-1) and second (episode-2) infection strains is shown in Table 1. Mutation analysis of the samples showed 15 mutations and 9 amino acid substitutions in the episode-1 strain and revealed distinct and a high number of mutations in the episode-2 strain. A total of 36 mutations were observed in the episode-2 strain: 18 were non-synonymous, 11 synonymous, and 1 upstream untranslated region (UTR), 1 downstream UTR, 3 deletions, and 2 stop codon mutations. There were 8 mutations identified within the RBD of the S protein in episode-2 strain.

**TABLE 1:** Comparison of mutations between the first episode (MW785206) and second episode (MW785207) of SARS-CoV-2 virus with reference strains Wuhan-Hu-1 (GenBank accession number NC_045512).

Gene region	Position (nt)	MW785206 (aa)	MW785207 (aa)
ORF 1a (266–13,441)	1,163	I,300F	–
	3,267	–	T1001I
	3,955	K1230N	–
	5,388	–	A1708D
	6,954	–	I2230T
	10,193	–	E3310K
	11,124	A3620V	–
	11,288–11,296	–	S3675^a^ G3676^a^ F3677^a^
ORF 1b (13,442–21,552)	14,120	–	P218L
	14,408	P314L	P314L
S (21,563–25,384)	21,766–21,771	–	H69^a^ V70^a^
	21,993–21,995	–	Y144^a^
	23,063	–	N501Y
	23,271	–	A570D
	23,604	P681R	P681H
	23,709	–	T716I
	24,506	–	S982A
	24,914	–	D1118H
ORF 3a (25,393–26,220)	25,690	G100C	-
ORF 8 (27,894–28,259)	27,972	–	Q27*^b^
	28,048	–	R52I
	28,095	–	K68*^b^
	28,111	–	Y73C
N (28,274–29,533)	28,280	–	D3L
	28,977	–	S235F

*The nucleotide and amino acid positions changes in the MW785206 and MW785207 strain, in comparison with the reference strain Wuhan-Hu-1 strain (NC_045512).*

*^a^Deletion.*

*^b^Stop codon.

*–, synonymous mutation; nt, nucleotide; aa, amino acid.*

## Discussion

Ever since the global emergence and spread of the major SARS-CoV-2 VOCs Alpha (B.1.1.7), Beta (B.1.351), Gamma (P.1), and Delta (B.1.617), uncertainty remains regarding the protective immunity of recovered patients to possible reinfections. Our genomic sequence data confirm the molecular evidence of SARS-CoV-2 reinfection with two different variants, as was reported from Hong Kong, Brazil, and the United Kingdom (Mulder et al., 2020; Adrielle Dos Santos et al., 2021; Nonaka et al., 2021; Tillett et al., 2021). The severity of episode-2 was more intense than episode-1, though he had taken his first shot of the Oxford–AstraZeneca COVID-19 vaccine (Covishield) on February 20, 3 days before testing positive for the second infection. Immuno-suppressive drugs like steroids can potentially delay the development of immunity following an infection, making the individual more susceptible to reinfection. However, the patient did not take steroids during or after episode-1 as his symptoms were mild. Other factors like older age and heart disease (hypertension) may have elevated the complications of episode-2 (SeyedAlinaghi et al., 2020). The mild infection during episode-1 likely failed to develop a strong and long-lasting immune response to the virus (Ni et al., 2020; Seow et al., 2020). However, we could not measure the immune status such as antibody titers because of the unavailability of blood samples. It is also possible that the immunity developed by the episode-1 strain was not sufficient to protect against the episode-2 strain, which was genetically different from the first one. Moreover, the RBD of episode-2 strain contained mutations (N501Y and deletions 69–70) which might confer a highly increased binding affinity to ACE2 receptors in human cells and alter the conformation of the N-terminal domain (NTD), enhancing the transmissibility of the SARS-CoV-2 (Kemp et al., 2020). In addition, the mutation P681H near the S1/S2 furin cleavage site with high variability has a unique and emerging characteristic that is important for membrane fusion and viral entry, which can increase the infectivity of SARS-CoV-2 (Yang et al., 2021). Phylogenetic analyses indicate that this lineage was spreading 40–70% more quickly than other lineages (Davies et al., 2021; Volz et al., 2021). In addition to N501Y, there is some evidence that the B.1.1.7 variant is unlikely to escape recognition by antibodies generated by prior infection or the vaccines (Collier et al., 2021; Muik et al., 2021). Interestingly, the non-synonymous mutation P218L and P314L in the nsp 12 RNA-dependent RNA polymerase gene may enhance viral entry and replication. We also identified numerous nonsense mutations. Of particular interest seems to be Q27* and K68* stop codons in open reading frame 8 (ORF8), which might play a role in immune evasion (Zhang et al., 2020; Zinzula, 2021). The lineage B.1.1.7 (Alpha) has been associated with evidence of increased hospitalization, mortality, transmissibility, severity, and possible immune evasion with potential implications for reinfection and vaccine effectiveness in patients (Pascall et al., 2021).

These findings concern whether the immunity acquired by natural infection or mass vaccination could confer adequate protection against the constantly evolving SARS-CoV-2. Therefore, continuous monitoring of genetic variations of SARS-CoV-2 strains is crucial for interventions such as vaccine and drug designs, treatment using monoclonal antibodies, and patient management.

## Data Availability Statement

The datasets presented in this study can be found in online repositories. The names of the repository/repositories and accession number(s) can be found below: https://www.ncbi.nlm.nih.gov/genbank/, MW785206 and https://www.ncbi.nlm.nih.gov/genbank/, MW785207.

## Ethics Statement

The studies involving human participants were reviewed and approved by the institutional research review board of icddr,b (protocol no. PR-20165). The patients/participants provided their written informed consent to participate in this study.

## Author Contributions

MEH designed the study, supervised the laboratory work, analyzed the data, and wrote the manuscript. MMR did laboratory tests and data analysis. MA, YK, MH, AH, and MMH did laboratory tests. MS and MC analyzed the clinical data and reviewed the manuscript. MZR reviewed the manuscript. MR conserved the study, reviewed the manuscript, and supervised the whole study. All authors contributed to the article and approved the submitted version.

## Conflict of Interest

The authors declare that the research was conducted in the absence of any commercial or financial relationships that could be construed as a potential conflict of interest.

## Publisher’s Note

All claims expressed in this article are solely those of the authors and do not necessarily represent those of their affiliated organizations, or those of the publisher, the editors and the reviewers. Any product that may be evaluated in this article, or claim that may be made by its manufacturer, is not guaranteed or endorsed by the publisher.

## Footnotes

1
https://www.worldometers.info/coronavirus/

2
https://pangolin.cog-uk.io/
